# Atopic Dermatitis Anti-IgE Paediatric Trial (ADAPT): the role of anti-IgE in severe paediatric eczema: study protocol for a randomised controlled trial

**DOI:** 10.1186/s13063-017-1809-7

**Published:** 2017-03-22

**Authors:** Susan Chan, Victoria Cornelius, Tao Chen, Suzana Radulovic, Mandy Wan, Rahi Jahan, Gideon Lack

**Affiliations:** 1 Guy’s and St. Thomas’ NHS Foundation Trust, Westminster Bridge Road, London, SE1 7EH UK; 2 King’s College London, King’s Health Partners, Asthma-UK Centre in Allergic Mechanisms of Asthma, Department of Asthma, Allergy and Respiratory Science, Guy’s Hospital, London, SE1 9RT UK; 3 Imperial Clinical Trials Unit, School of Public Health, Imperial College London, Stadium House, 68 Wood Lane, London, W12 7RH UK; 4 Department of Clinical Sciences, Liverpool School of Tropical Medicine, Liverpool, L3 5QA UK

**Keywords:** Eczema, Paediatric, Atopic dermatitis, Anti-IgE, Omalizumab, Randomised controlled trial, Double blind, Placebo, Xolair®

## Abstract

**Background:**

The evidence for systemic treatments for severe childhood eczema is limited and largely based on extrapolation of data from adult studies. Current therapies are often immunosuppressant and may be associated with both short- and long-term side effects. There is increasing in vitro and murine-model evidence for the role of IgE in the immunopathogenesis of atopic eczema. The aim of the study is to assess whether anti-IgE treatment (omalizumab) improves eczema, compared to placebo.

**Methods/design:**

The Atopic Dermatitis Anti-IgE Paediatric Trial (ADAPT) is a randomised, double-blind, placebo-controlled study assessing the role of anti-IgE in the management of severe paediatric eczema. Children with severe atopic eczema, with an objective SCORing Atopic Dermatitis (SCORAD) score of over 40 will be recruited. These children are candidates for systemic therapy, have failed systemic therapy or have experienced side effects from systemic therapy. Sixty-two patients aged between 4 and 19 years will receive anti-IgE for 6 months. The primary outcome measure will be the validated eczema score, the objective SCORAD at 24 weeks. This study has 90% power to detect a 33% relative reduction in SCORAD between active and placebo groups, with 5% significance.

**Discussion:**

IgE may have a role to play in eczema, particularly in childhood. This forms the basis for the hypothesis that anti-IgE may be an effective treatment in this patient population.

This will be the largest study to evaluate the efficacy of anti-IgE (omalizumab) versus placebo in children with severe eczema. The findings will help to clarify the role of anti-IgE as a potential treatment option in patients with severe childhood eczema.

**Trial registration:**

European Clinical Trials Database (EudraCT) Number: 2010-020841-29. Assigned on 14 May 2010.

ISRCTN Registry, Identifier: ISRCTN15090567. Retrospectively assigned on 3 December 2014.

ClinicalTrials.gov, Identifier: NCT02300701. First received 21 November 2014.

**Electronic supplementary material:**

The online version of this article (doi:10.1186/s13063-017-1809-7) contains supplementary material, which is available to authorized users.

## Background

Atopic eczema (AE) (or atopic dermatitis) is an inflammatory, chronically relapsing, pruritic skin disease. The prevalence in the United Kingdom (UK) is 16% for 6 to 7-year-olds (with 2.2% classed as severe) and 10.6% for 13 to 14-year-olds (with 1.5% classed as severe) [1]. AE is associated with a significant economic and psychosocial burden.

A complex interaction between genetics, environment and immunology define the pathophysiology of AE. There is increasing in vitro and murine-model evidence for the role of IgE in the immunopathogenesis of atopic dermatitis, with higher IgE levels linked with more severe disease [2, 3]. IgE is likely to be of more relevance in paediatric disease than in adult disease, where AE is thought to become less allergen-driven and more ‘autoreactive’ [4]. This study focusses on a paediatric atopic population, to target patients in whom IgE is more likely to be relevant.

The management of AE includes the identification and elimination of trigger factors, appropriate use of topical treatments, and adequate patient education. When systemic therapy is required, systemic immunosuppressants, such as ciclosporin, azathioprine, and methotrexate, have been used [5]. However there is limited published evidence in childhood practice to guide clinical use. In addition, most of these drugs are not licensed for use in patients with severe AE in many countries. With the potential for serious side effects, these drugs are often not recommended for long-term use.

Omalizumab (Xolair®, Novartis) is the only commercially available anti-IgE antibody. It binds to human IgE, limiting mast cell degranulation and inhibiting the release of inflammatory mediators. It is licensed in the European Union for use in chronic spontaneous urticaria from the age of 12 years, and severe persistent allergic asthma from the age of 6 years [6]. Safety data suggests that Xolair® (omalizumab) is well tolerated in children [7].

Our hypothesis is that anti-IgE will reduce the levels of IgE in children with severe AE and alleviate their symptoms.

### Objectives

#### Primary objectives

1. To determine whether the intervention is associated with an improvement in eczema severity, compared to placebo.

#### Secondary objectives

1. To evaluate whether the intervention is associated with a change in eczema severity, eczema quality of life and coexisting allergic disease
2. To assess whether it is safe and well tolerated in eczema
3. To investigate whether the intervention is associated with fewer eczema exacerbations and infective episodes of eczema
4. To determine the change in reactivity to allergens

## Methods/design

### Study design

The Atopic Dermatitis Anti-IgE Paediatric Trial (ADAPT) is a randomised, double-blind, placebo-controlled study. It uses a hub and spoke method of recruitment where treatment will be delivered at a single centre, but recruitment will occur from hospitals in and around London, United Kingdom. The trial will test the superiority of anti-IgE (omalizumab) versus placebo in the treatment of severe childhood eczema. The protocol was designed in accordance with the SPIRIT (Standard Protocol Items: Recommendations for Interventional Trials) guidelines for interventional trials (Additional file 1: Figure S1; and Additional file 2: Figure S2).

Sixty-two children, aged 4–19 years, with severe eczema will be enrolled in the study which is based at Guy’s and St. Thomas’ NHS Foundation Trust, London, United Kingdom.

### Study population

The study population will be children with severe AE, who are candidates for systemic therapy for AE. Children will also be eligible if they have failed systemic therapy, or have experienced side effects from systemic therapy.

### Inclusion criteria

1. Aged 4–19 years
2. Severe eczema with (1) an objective SCORing Atopic Dermatitis (SCORAD, a validated eczema severity score) score of over 40, which is (2) unresponsive to optimal topical therapy (potent topical steroids and/or topical calcineurin inhibitors) or systemic therapy, with (3) no impression of lack of compliance, (4) a (Children’s) Dermatology Life Quality Index Questionnaire ((C)DLQI) score of ≥10 and (5) where active skin infection has been ruled out and/or adequately treated
3. A raised specific IgE (SpIgE) (>0.35 IU/ml or kUA/L) or skin-prick test (SPT) (>3 mm) to at least one food allergen or one aeroallergen *and/or*
4. The clinical impression that allergic exposures cause worsening eczema
5. Total IgE level >300 IU/ml or kU/L
6. Clinically proven IgE-mediated allergic disease including at least one of the following: (1) immediate hypersensitivity to a food as proven by raised SpIgE or SPT greater than the 95% positive predictive value or ≥8 mm, or a positive double-blind, placebo-controlled food challenge, (2) allergic rhinoconjunctivitis as defined by sensitisation to a respiratory allergen and a clinical history of rhinoconjunctivitis symptoms when exposed to the relevant allergen, (3) allergic asthma: a history of a cough, wheeze or shortness of breath that (a) was responsive to therapy with bronchodilators on two or more occasions in the previous 24 months, (b) required one visit to a physician in the previous 24 months and (c) occurred during the night, during early morning or upon exercising in the intervals between exacerbations at any time in the previous 12 months and (d) where allergic exacerbations can be clinically related to an allergen exposure *with* a corresponding positive SPT or SpIgE to the allergen
7. Written informed consent to participate, and assent if appropriate

### Exclusion criteria

Children will not be able to participate if (1) they and/or their families are unable to comply with the regime of two to four weekly injections and clinic visits, (2) there is evidence of underlying immune compromise, autoimmune disease, immune complex-mediated conditions, (3) there is malignancy or a history of malignancy, (4) there is a known cardiovascular or ischaemic cerebrovascular abnormality, (5) there is other serious or uncontrolled systemic disease, (6) the subject is pregnant or lactating, (7) there is a known history of hypersensitivity or anaphylaxis to anti-IgE injections or its constituents; (8) there is insufficient understanding of the trial assessments, (9) they have participated in a clinical trial of an investigational medicinal product (CTIMP) in the previous 60 days or (if known) four half-lives of the medication under investigation, whichever is the greater. In this case, entry may be delayed until the appropriate time or (10) the investigator feels that there is a good clinical reason why the child would be unsuitable for the study.

### Recruitment

Sixty-two children with severe AE, will be enrolled into the study which is based at Guy’s and St. Thomas’ NHS Foundation Trust. A hub and spoke method of recruitment will be employed. Referring hospitals from in and around London will be able to refer patients to the study team. Recruitment will be open to participants in the UK who are willing to travel to the central treatment centre for their treatment and follow-up visits.

### Intervention

Each participant will be enrolled for 48 weeks, consisting of a 24-week treatment period and a 24-week follow-up period. A detailed timeline of the study is shown in the Gantt chart in Additional file 3: Figure S3.

The active treatment is omalizumab (Xolair®) injection, manufactured by Novartis and provided as 150-mg single-use vial. The manufacturer also provided the comparator. Placebo is formulated to be comparable in appearance to the omalizumab and contains all of the same excipients except the drug substance. The dose and dosing frequency of omalizumab (Xolair®) or placebo will be determined by baseline total IgE (range: 30 to 1500 IU/ml), measured before treatment commences, and body weight (kg) at the randomisation visit. The dose of omalizumab (Xolair®) will be calculated using the latest manufacturer’s dosing tables (according to the current Summary of Product Characteristics) [8]. The dose of Xolair® (omalizumab) stated on the table, closest to that child’s weight and IgE levels, is used. Doses are 75, 150, 225, 300, 450, 525 or 600 mg every 2 or 4 weeks. The equivalent volume for placebo doses will be calculated in the same way. Participants will remain on the same dose throughout the 24 weeks of treatment. Treatment is administered subcutaneously in the deltoid region or the thigh as one to four injections depending on the dose. Anaesthesia using topical local anaesthetic (lidocaine cream) or ethyl chloride spray can be used prior to the injection, according to patient preference.

### Randomisation and blinding

Participants who consent to participate and who fulfil the eligibility criteria are randomly allocated to receive either omalizumab (Xolair®) or placebo in a 1:1 allocation ratio using a secure web-based randomisation system. The allocation sequence is computer-generated by the UK Clinical Research Collaboration (UKCRC)-registered King’s College London Clinical Trials Unit in conjunction with an independent statistician. Participants are allocated to treatment group using minimisation with variables IgE (≤1500, >1500 IU/ml) and age (below 10 years or 10 years and older).

Blinding is maintained by strictly adhering to established procedures to maintain separation between staff who take outcome measurements and staff who deliver the treatment. All study team physicians, researchers and research nurses involved in primary outcome assessments, as well as participants and their families, are blinded to treatment allocation until the primary analysis is completed. To ensure that these individuals remain blinded to treatment allocation, randomisation details are sent electronically to the independent pharmacy team, and the preparation and administration of the treatment will be restricted to a group of trained clinical staff. Unblinded clinical staff are assigned to collect and then return used vials of active or placebo to pharmacy. Preparation and administration of treatment by unblinded staff will occur in a closed treatment room, separate from the main clinical area. Entry to this room is barred to blinded staff during the preparation and administration of the treatment by unblinded staff. Unblinded clinical staff will not be involved in trial-related primary outcome assessments to minimise the risk of disclosing the allocation of treatment. Staff members who obtain outcome measurements are not involved in any of the steps involving handling of the intervention.

All patients will be provided with an emergency card with relevant contact details to be carried for the duration of the trial. Code break will be provided in clinically relevant situations to clinicians, by the pharmacy department.

The study statistician will analyse the data blinded to treatment allocation.

### Concomitant medications

Participants may continue to take their conventional treatments for AE during the course of the study. Concomitant AE medications would include topical treatments including any emollients, bath additives, topical steroids, topical calcineurin inhibitors, wet wraps and systemic treatment.

During treatment with omalizumab (Xolair®)/placebo, the participants/parents will monitor the child’s AE at home and, if there is any deterioration, this would trigger the participants/parents to contact the study team to discuss drug doses or a reassessment and modification of therapy. An exacerbation will be identified and managed, and any additional therapy or changes in doses of existing treatment will be recorded.

Any medication required for any ongoing illness, contraception and any rescue medications will also be permitted and recorded. Female patients who have attained menarche will undergo a pregnancy test at the baseline and randomisation visits.

### Outcomes

Details of the primary and secondary outcomes of the study are summarised in Additional file 4: Table S1.

### Primary outcome

#### Objective SCORAD

This will be based upon the assessment of the objective SCORAD (SCORing Atopic Dermatitis) score after 24 weeks of treatment. Further assessments at 36 and 48 weeks (post-treatment reviews) will be performed.

The objective SCORing Atopic Dermatitis (eczema severity score) is a validated clinical tool used to assess the extent and severity of eczema [9]. It evaluates six clinical characteristics to determine disease severity: (1) erythema, (2) edema/papulation, (3) oozing/crust, (4) excoriation, (5) lichenification and (6) dryness. The maximum score is 83.

### Secondary outcomes

#### Treatment failure

Treatment failure will be defined in patients who, after the first 12 weeks of treatment, have persistent severe eczema despite two courses of rescue therapy with oral prednisolone (0.5 to 1 mg/kg/day of oral prednisolone for a week at a maximum dose of 40 mg/day, followed by a week at 50% of this dose).

### Alternative systemic therapy

An assessment will be made of patients in whom (1) alternative systemic therapy has been started as a result of treatment failure as defined above or (2) where alternative systemic therapy is started after 12 weeks, and by 30 weeks.

### Eczema quality of life

This will be assessed by the Patient-oriented Eczema Measure Questionnaire (POEM) and the (Children’s) Dermatology Life Quality Index Questionnaire (C)DLQI.

The POEM is a validated, patient-derived assessment measure for monitoring atopic eczema severity over the past week [10]. The questionnaire has seven items, each with a 5-point scale from no days to every day. The scores range from 0 to 28. The data will be collected at baseline, 4-weekly during treatment up to 24 weeks, and at 36 and 48 weeks.

The (C)DLQI is a validated questionnaire designed to measure the condition of the participant’s skin over the past week [11]. The questionnaire has 10 questions and the total score ranges from 0 to 30 [12]. The information will be followed up at baseline, 4-weekly during treatment up to 24 weeks, and at 36 and 48 weeks.

### Eczema severity

This will be assessed by the objective and subjective SCORAD scores, and the Eczema Area and Severity Index (EASI).

The subjective SCORAD score is an add-on score to the objective SCORAD score, which also assesses subjective symptoms of pruritus and sleep loss [13], each on a scale of 0 to 10 [13]. The subjective score adds up to an additional 20 points to the objective SCORAD score.

The EASI is an investigator-assessed instrument to measure the extent (area) and severity of atopic eczema [14]. The severity of four features (erythema, edema/papulation, excoriation and lichenification) are scored from 0 to 3 (none, mild, moderate and severe, respectively) with half-steps allowed, in a representative patch of eczema in four regions (head/neck, upper limbs, trunk, lower limbs). The percentage area affected is calculated. The EASI scores range from 0 to 72, and higher scores represent more severe eczema.

### Effect on coexisting allergic disease

This will be assessed by the Paediatric Allergic Disease Quality of Life Questionnaire (PADQLQ), a measure of health-related quality of life that encompasses effects of allergic conditions on a child’s or teenager’s eyes, ears, nose, lungs, skin, emotions, and everyday activities over the past week [15]. The PADQLQ contains 26 questions, with a 7-point scale from ‘not troubled’ to ‘extremely troubled’.

### Number of eczema exacerbations

The number of eczema exacerbations will be recorded. Exacerbations will be defined as clinician-diagnosed exacerbation of eczema *or* increase in the SCORAD score by 15 points from the last recorded SCORAD score with patient/parent/guardian perception of worsening eczema.

### Infective episodes of eczema

Infective episodes of eczema will be defined as clinician-diagnosed and treated infective episode of eczema, *or* clinically apparent, culture-positive infective exacerbations.

### Total and allergen-specific IgE (SpIgE)

This will be determined at screening and at 24 weeks.

### Reactivity to food and aeroallergens

The SPT is an assessment of the allergic response to specific food and aeroallergens. The SPT introduces a tiny amount of allergen into the skin, eliciting a small, localised allergic response in the form of a wheal and flare at the site of testing [16, 17].

### Steroid cream usage

The amount of potent steroid creams and calcineurin inhibitors used will be assessed. Tubes of creams are weighed at each visit using digital scales. The frequency of use per day and per week since the last visit, and the body surface area covered, will be recorded.

### Data collection and management

Data will be collected on source data worksheets and stored in the patient’s medical notes, and will be transcribed to the electronic Case Report Form (eCRF) system. The eCRF will be designed and maintained by the King’s Clinical Trials Unit using the InferMed MACRO system (Version 4.0), which is compliant with Good Clinical Practice (GCP) and 21 CRF Part 11. At the end of the study, the eCRF system will be locked and data exported for final analysis. Patient data will be anonymised. All anonymised data will be stored on a password-protected computer. All trial data will be stored and archived in line with the Medicines for Human Use (Clinical Trials) Amended Regulations 2006 as defined in the King’s Health Partners Clinical Trials Office Archiving Standard Operating Procedure.

### Statistics

#### Sample size

Omalizumab is administered at 2- to 4-weekly intervals, by subcutaneous injection, at a suitable health care facility. It is available in the UK under a negotiated patient access scheme for asthma and chronic urticaria. A reasonable treatment benefit would be required for omalizumab to be adopted into practice. We aim to detect a 33% relative reduction in SCORAD scores. In order to detect a change in the SCORAD score of 13.5 points between the treatment arms, assuming an SD of 15 [18] with 5% significance and 90% power, and incorporating a 15% dropout rate, we aim to recruit 62 participants (31 to each arm).

### Analysis

A Consolidated Standards of Reporting Trials (CONSORT) flow chart will be constructed and will include the number of eligible patients, number of patients agreeing to enter the trial, number of participants withdrawing and the number included in the analyses [19].

Baseline characteristics will be summarised and tabulated by treatment arm. The analysis will follow an intention-to-treat (ITT) principle and participants will be analysed in the arm to which they were allocated regardless of subsequent treatment received. The number lost to follow-up will be tabulated by treatment arm and visit. The proportions of participants missing objective SCORAD values will be summarised in each arm and at each time point. The baseline characteristics (age, gender, objective and subjective SCORAD, Body Mass Index (BMI), asthma (Y/N), food allergy (Y/N), rhinoconjunctivitis (Y/N) and referral source (self-referred/tertiary) of those missing follow-up will be compared to those with complete follow-up. The recorded reasons for withdrawal from the trial will be summarised [20].

A linear mixed model will be used to obtain an estimate for the mean difference in objective SCORAD scores between the two treatment groups. Participant will be included as a random intercept (investigating adding a random slope on time), time (investigating the possibility of linearising this effect across 8, 12, 16, 20 and 24 weeks), time-by-group interaction, baseline objective SCORAD score, IgE (≤1500, >1500 IU/ml) and age (below 10 years or 10 years and older) as fixed effect. An overall treatment effect for the objective SCORAD score at 24 weeks will be estimated. A number of sensitivity analyses will be performed to estimate the treatment effect adjusting for use of topical steroids and alternative systemic therapy. A complier average causal effect (CACE) will be performed where ‘complier’ will be defined as a participant who completes more than 50% of injections [21].

Secondary analysis will be based on the ITT population and primarily focussed on the outcome at week 24. Subjective SCORAD scores will be analysed in a similar manner as for objective SCORAD scores. For all other outcomes we will only adjust for the minimisation variables IgE (≤1500, >1500 IU/ml), age (below 10 years or 10 years and older) and baseline data (as appropriate). Binary outcomes will be analysed using a logistic regression model and count outcomes using Poisson regression, negative binomial regression or zero-inflated Poisson, as appropriate, and continuous outcomes using a linear regression model.

Adverse events will be tabulated separately by type (adverse events, adverse reaction, unexpected adverse reaction, serious adverse event, serious adverse reaction or unexpected serious adverse reactions). These will be summarised overall (0–48 weeks) and the time of occurrence will be explored by randomised intervention where appropriate.

A detailed statistical analysis plan has been developed in conjunction with the protocol and is available [Reference from *Trials* if accepted].

## Discussion

The management of AE necessitating systemic therapy is currently an area of intense interest. When systemic therapy is required in childhood AE, systemic immunosuppressants have been used with limited evidence, and in many cases as an unlicensed indication [5].

There is increasing evidence for the role of IgE in the immunopathogenesis in AE. Omalizumab (Xolair®, Novartis) is the only commercially available anti-IgE antibody. ADAPT is the largest randomised, double-blind, placebo-controlled study to date evaluating anti-IgE (omalizumab) in severe childhood AE. The aim is to definitively assess the role of anti-IgE as an alternative systemic treatment for AE.

### Trial status

The ADAPT study received favourable ethical opinion on 7 July 2011 and local R&D approvals were obtained on 11 November 2014. Participant recruitment started on 20 November 2014 and is ongoing.

## Additional files

Additional file 1: Figure S1. SPIRIT 2013 Checklist. (DOC 272 kb)

Additional file 2: Figure S2. Schedule of enrolment, interventions, and assessments. (DOC 68 kb)

Additional file 3: Figure S3. Gantt chart (timeline of the study, DOC). (DOCX 16 kb)

Additional file 4: Table S1. Primary and secondary outcomes (primary and secondary outcome measures of the study). (DOCX 20 kb)

## Abbreviations

(C)DLQI: (Children’s) Dermatology Life Quality Index
AD: Atopic dermatitis
ADAPT: Atopic Dermatitis Anti-IgE Paediatric Trial
AE: Atopic Eczema
CTIMP: Clinical trial of an investigational medicinal product
EASI: Eczema Area and Severity Index
eCRF: Electronic Case Report Form
IgE: Immunoglobulin E
PADQLQ: Paediatric Allergic Disease Quality of Life Questionnaire
POEM: Patient Oriented Eczema Measure
REC: Research Ethics Committee
SCORAD: SCORing Atopic Dermatitis
SPT: Skin-prick test
