# Supplementary material for: Atopic Dermatitis Anti-IgE Paediatric Trial (ADAPT): the role of anti-IgE in severe paediatric eczema: study protocol for a randomised controlled trial
Source: Trials. 2017 Mar 22;18:136. doi: 10.1186/s13063-017-1809-7 (PMC5361704; doi:10.1186/s13063-017-1809-7)
Supplement: Supplementary file 2 — Schedule of enrolment, interventions, and assessments. (DOC 68 kb) [file 13063_2017_1809_MOESM2_ESM.doc]

Figure 2. Schedule of enrolment, interventions, and assessments.

|  | **STUDY PERIOD** | | | | | | | | | | | | | | |
| --- | --- | --- | --- | --- | --- | --- | --- | --- | --- | --- | --- | --- | --- | --- | --- |
|  | **Enrolment** | **Allocation** | **Post-allocation** | | | | | | | | | | | **Follow up** | |
| **TIMEPOINT**** | ***-t_1_*** | **0/*t_1_*** | ***t_2*_*** | ***t_4_*** | ***t_6_*** | ***t_8_*** | ***t_10*_*** | ***t_12_*** | ***t_14*_*** | ***t_16_*** | ***t_18*_*** | ***t_20_*** | ***t_22*_*** | ***t_24_*** | ***t_36/48_*** |
| **ENROLMENT:** |  |  |  |  |  |  |  |  |  |  |  |  |  |  |  |
| **Eligibility screen** | X |  |  |  |  |  |  |  |  |  |  |  |  |  |  |
| **Informed consent** | X |  |  |  |  |  |  |  |  |  |  |  |  |  |  |
| **Bloods** | X |  |  |  |  |  |  |  |  |  |  |  |  | X |  |
| **Skin swabs** | X |  |  |  |  |  |  |  |  |  |  |  |  |  |  |
| **Urinanalysis** | X |  |  |  |  |  |  |  |  |  |  |  |  | X | X |
| **Skin prick tests** | X |  |  |  |  |  |  |  |  |  |  |  |  | X |  |
| **Allocation** |  | X |  |  |  |  |  |  |  |  |  |  |  |  |  |
| **INTERVENTIONS:** |  |  |  |  |  |  |  |  |  |  |  |  |  |  |  |
| **Anti-IgE** |  |  |  |  |  |  |  |  |  |  |  |  |  |  |  |
| **Placebo** |  |  |  |  |  |  |  |  |  |  |  |  |  |  |  |
| **ASSESSMENTS:** |  |  |  |  |  |  |  |  |  |  |  |  |  |  |  |
| **Quality-of-Life Questionnaires** | X | X |  | X |  | X |  | X |  | X |  | X |  | X | X |
| **SCORAD** | X | X |  | X |  | X |  | X |  | X |  | X |  | X | X |
| **EASI** | X | X |  | X |  | X |  | X |  | X |  | X |  | X | X |
| **Height/Weight** | X | X |  |  |  |  |  |  |  |  |  |  |  | X | X |
|  |  |  |  |  |  |  |  |  |  |  |  |  |  |  |  |
