# Supplementary material for: Atopic Dermatitis Anti-IgE Paediatric Trial (ADAPT): the role of anti-IgE in severe paediatric eczema: study protocol for a randomised controlled trial
Source: Trials. 2017 Mar 22;18:136. doi: 10.1186/s13063-017-1809-7 (PMC5361704; doi:10.1186/s13063-017-1809-7)
Supplement: Supplementary file 3 — Gantt chart (timeline of the study, DOC). (DOCX 16 kb) [file 13063_2017_1809_MOESM3_ESM.docx]

**Figure 3: Gantt chart**
