# Supplementary material for: Atopic Dermatitis Anti-IgE Paediatric Trial (ADAPT): the role of anti-IgE in severe paediatric eczema: study protocol for a randomised controlled trial
Source: Trials. 2017 Mar 22;18:136. doi: 10.1186/s13063-017-1809-7 (PMC5361704; doi:10.1186/s13063-017-1809-7)
Supplement: Supplementary file 4 — Primary and secondary outcomes (primary and secondary outcome measures of the study). (DOCX 20 kb) [file 13063_2017_1809_MOESM4_ESM.docx]

| **Outcome** | **Endpoints** |
| --- | --- |
| **Primary** |  |
| Objective SCORAD | Difference in the objective SCORAD in both groups after 24 weeks of treatment |
| **Secondary** |  |
| Treatment failure | Participants who have persistent severe eczema despite 2 courses of rescue therapy (0.5 to 1mg/kg/day of oral prednisolone for a week at a maximum dose of 40mg/day, followed by a week at 50% of this dose) |
| Alternative systemic therapy | Requirement for alternative systemic therapy |
| Eczema quality of life | - POEM - (C)DLQI |
| Eczema severity | - Objective and Subjective SCORAD - EASI score |
| Effect on co-existing allergic disease | PADQLQ |
| Number of eczema exacerbations^c^ | - Clinician diagnosed exacerbation of eczema or - Increase on SCORAD by 15 points from last recorded SCORAD with participants/parent perception of worsening eczema |
| Infective episodes of eczema^c^ | Clinician diagnosed and treated infective episode of eczema, or clinically apparent, culture positive infective exacerbations |
| IgE^a^ | Change in total and allergen specific IgE |
| Reactivity to food and aeroallergens^a^ | Change in skin prick test reactivity to food and aeroallergens |
| Medication usage | The amount of potent steroid creams and calcineurin inhibitors used. |
| **Safety** |  |
| Adverse events^b^ | Spontaneous reported AE will be collected throughout the follow-up period |

**Table 1: Primary and Secondary outcomes**

^a^ Only collected at screening and 24 weeks of treatment. The remaining outcomes are collected at baseline, 4 weekly during the 24 weeks of treatment, 36 weeks and 48 weeks.

^b^ Blood test and urine samples will be collected at baseline, 24 weeks, 36 weeks, and 48 weeks. Clinical observations will be examined at every visit.

^c^ Chi‐Square goodness‐of‐fit tests will be used to select the suitable model.

SCORAD: SCORing Atopic Dermatitis; PADQLQ: Paediatric Allergic Disease Quality of Life Questionnaire; (C)DLQI: (Children’s) Dermatology Life Quality Index; EASI: Eczema Area and Severity Index; AE: Adverse events; POEM: Patient-oriented Eczema Measure.
